# Characteristics and prognostic analysis of simultaneous bilateral sudden sensorineural hearing loss

**DOI:** 10.3389/fneur.2023.1179579

**Published:** 2023-05-05

**Authors:** Yingjun Wang, Wenping Xiong, Xiao Sun, Fujia Duan, Kunpeng Lu, Haibo Wang, Mingming Wang

**Affiliations:** ^1^Department of Otology Medicine, Shandong Provincial ENT Hospital, Shandong University, Jinan, China; ^2^Shandong Institution of Otolaryngology, Jinan, China; ^3^Department of Otology Center, Shandong Provincial ENT Hospital, Shandong University, Jinan, China

**Keywords:** sudden sensorineural hearing loss, bilateral, simultaneous, propensity score, prognosis

## Abstract

**Objective:**

To evaluate the clinical characteristics of simultaneous bilateral sudden sensorineural hearing loss (Si-BSSNHL) as well as its prognostic factors.

**Methods:**

Patients with Si-BSSNHL who were admitted to the Department of Otology Medicine between December 2018 and December 2021 were enrolled in the case group. Propensity score matching (PSM) for sex and age was used to select the control group, which included people who had unilateral sudden sensorineural hearing loss (USSNHL) during the same time period. Hearing recovery, audiological examinations, vestibular function assessments, laboratory tests, and demographic and clinical manifestations were analyzed for intergroup comparisons. Binary logistic regressions were used for both univariate and multivariate analyses of Si-BSSNHL prognostic factors.

**Results:**

Before PSM, the Si-BSSNHL and USSNHL groups differed significantly (*p* < 0.05) in terms of time from onset to treatment, initial pure-tone average (PTA), final PTA, hearing gain, audiogram curve type, proportion of tinnitus, high-density lipoprotein level, homocysteine level, and effective rate. After PSM, significant differences were also observed in time from onset to treatment, initial PTA, final PTA, hearing gain, total and indirect bilirubin levels, homocysteine level, and effective rate between the two groups (*p* < 0.05). There was a significant difference in the classification of therapeutic effects between the two groups (*p* < 0.001). For prognostic analysis, the audiogram curve type was significantly different between the effective group and the ineffective groups of Si-BSSNHL (*p* = 0.01), in which the sloping type was an independent risk factor for the prognosis of the right ear in Si-SSNHL (95% confidence interval, 0.006–0.549, *p* = 0.013).

**Conclusion:**

Patients with Si-BSSNHL had mild deafness, elevated total and indirect bilirubin and homocysteine levels, and poorer prognosis than those with USSNHL. Audiogram curve type was linked to the therapeutic effect of Si-BSSNHL, and the sloping type was an independent risk factor for a poor prognosis in the right ear of Si-SSNHL.

## 1. Introduction

Sudden sensorineural hearing loss (SSNHL) is a sudden, unexplained sensorineural hearing loss of ≥20 dB HL in at least two adjacent frequencies that occurs within 72 h (1). The incidence of SSNHL is approximately 5–30 per 100,000 people per year, and 95% of SSNHL cases are unilateral (2). Although the incidence of bilateral SSNHL (BSSNHL) is much lower than that of unilateral SSNHL (USSNHL), the incidence of BSSNHL has been increasing annually, accounting for 4.9–8.6% (3, 4). BSSNHL can be divided into simultaneous BSSNHL (Si-BSSNHL) depending on how the disease is progressing: sudden hearing loss in both ears simultaneously or within 3 days, and sequential BSSNHL (Se-BSSNHL): sudden hearing loss in both ears at an interval of more than 3 days (5). The rapid onset of Si-BSSNHL has a significant negative impact on patients’ quality of life and social functions, and should be taken more seriously by physicians. In previous studies, there were few cases of Si-BSSNHL, confounding factors were rarely considered, fewer indicators were analyzed, and the results were controversial (5, 6). In this study, we set up a sex-and age-matched control group for USSBHL while conducting an exhaustive analysis of the relevant factors affecting the prognosis of Si-BSSNHL.

## 2. Materials and methods

### 2.1. Participants

Patients with Si-BSSNHL who were admitted to the Department of Otology between December 2018 and December 2021 were included in the case group, and patients with USSNHL during the same time period were selected as the control group. The inclusion criteria of case group were as follows: bilateral sudden deafness of ≥20 dB HL in at least two adjacent frequencies that occurs simultaneously or sequentially involving both ears within 72 h; age > 18 years; and first onset and duration ≤30 days. The inclusion criteria of control group were as follows: unilateral idiopathic sudden deafness (1); age > 18 years; and first onset and duration ≤30 days. Exclusion criteria of both groups were middle ear lesions, Meniere’s disease, drug poisoning, noise-induced deafness, trauma, post-cochlear lesions, autoimmune diseases, and neurological, infectious, or hematologic diseases.

### 2.2. Ethics statement

This study was carried out in accordance with the principles of the Declaration of Helsinki and approved by the ethics committee of our hospital (XYK20180605). Because this was a retrospective study, the need for informed consent was waived.

### 2.3. Research method

#### 2.3.1. Data collection

Data on patient sex, age, time from onset to treatment, combined diseases (hypertension, diabetes mellitus, and coronary heart disease), accompanying symptoms (vertigo, tinnitus, aural fullness, and counted by person), audiological examinations, vestibular function assessments, and laboratory tests (including metabolic factors, inflammatory factors, and coagulation indexes) were collected.

#### 2.3.2. Audiological examinations and efficacy assessments

Pure tone audiometry (GSI-61, United States), acoustic immittance (GSI Tympstar, United States), distortion product otoacoustic emission (IHS Smart EP, United States), and auditory brainstem response (IHS Smart EP, United States) were all used to rule out middle ear and retro-cochlear lesions. Audiogram curve type was classified as ascending, sloping, flat, or total deafness. The mean hearing threshold at 500 Hz, 1 kHz, 2 kHz, and 4 kHz was used to calculate pure tone average (PTA), and the degree of deafness was categorized according to the severity of the hearing loss: 25–40 dB HL as mild, 41–60 dB HL as moderate, 6 l–80 dB HL as severe, and > 80 dB HL as profound. The initial PTA was the pure-tone hearing threshold audiometric result examined before treatment in our hospital after the onset of the disease, and the final PTA was the hearing threshold result at 30 days post treatment.

The efficacy evaluations were divided into complete recovery: the hearing frequency returned either to the healthy side or normal level, or to the level before the disease started; partial recovery: the hearing frequencies improved by more than 30 dB on average; slight recovery: the hearing frequencies improved by 15–30 dB on average; and no recovery: the hearing frequencies improved by less than 15 dB on average. Complete, partial, and slight recovery were all included in the effective group. Patients with Si-BSSNHL were categorized as ineffective if both ears were ineffective and as effective if one or both ears were effective.

#### 2.3.3. Vestibular function assessments

Vestibular function assessments included the caloric test (Ulmer VNG, v. 1.4; SYNAPSYS, Marseille, France), video head impulse test (Ulmer, Synapsys, Marseille, France), vestibular evoked myogenic potential (o/cVEMP) test (Neurosoft LTD, Ivanov, Russia), and vestibular autorotation test (Western Systems Research, Pasadena, United States). Any abnormality in vestibular function assessments is considered as positive.

#### 2.3.4. Laboratory tests and imaging assessments

Routine peripheral blood samples were collected on the morning of the second day after admission. The metabolic indices included total bilirubin (TBIL), indirect bilirubin (IBIL), superoxide dismutase (SOD), glucose (GLU), triglyceride (TG), total cholesterol (TC), high-density lipoprotein (HDL), low-density lipoprotein (LDL), and homocysteine (Hcy). Inflammatory factors and coagulation parameters include C-reactive protein (CRP), neutrophil count, lymphocyte count, monocyte count, platelet count, and fibrinogen. The ratios of neutrophils to lymphocytes (NLR), monocytes-lymphocytes (MLR), and platelets-lymphocytes (PLR), respectively, are defined as the ratios of neutrophils, monocytes, and platelets to lymphocytes, respectively.

Contrast-enhanced MRI (PHILIPS, Intera, Holland) were performed to exclude occupying lesions.

#### 2.3.5. Treatment

During hospitalization, all patients received the following treatment: improvement of microcirculation (*Ginkgo biloba* extract), glucocorticoids (methylprednisolone sodium succinate), reduction of fibrinogen (batroxobin), and neurotrophic (methylcobalamin or mouse nerve growth factor) drugs. Patients with hypertension or diabetes mellitus were treated symptomatically and administered methylprednisolone sodium succinate systemically or locally behind the ear (in case of poor glycemic or blood pressure control), depending on the patient’s glucose or blood pressure level.

### 2.4. Statistical analysis

A 1: 1 nearest neighbor matching was performed for sex and age between the case and control groups using propensity score matching (PSM), and the caliper was set at 0.05. For normally distributed variables, continuous variables are shown as mean ± standard deviation, and comparisons between groups were performed using an independent sample *t*-test. For non-normally distributed variables, the median (interquartile range) was used for continuous data, and the non-parametric Mann–Whitney *U* test was used to compare groups. The categorical variables were compared using the Chi-square and Fisher’s exact tests. Binary logistic regression analysis was applied for univariate and multivariate analysis, and differences were significant at *p* < 0.05. The statistical software package SPSS 23.0 (IBM Corp., Armonk, NY, United States) was used for all analyses.

## 3. Results

### 3.1. Clinical data before PSM

A total of 50 cases of Si-BSSNHL were included in the case group, with a median age of 55.5 years (26 males and 24 females). Before PSM, the control group included 189 USSNHL cases with a median age of 47 years (100 males and 89 females). The differences in age, hearing gain, time from onset to treatment, initial PTA, final PTA, audiogram curve type, proportion of tinnitus, HDL and Hcy levels, and the effective rate were significant (*p* < 0.05) between the case group and control group, as shown in Table 1.

**Table 1:** Demographics and clinical characteristics of patients (Before PSM).

Variable	Si-BSSNHL (*n* = 50)	USSNHL (*n* = 189)	Statistics	Value of *p*
Age of onset	55.5 (39, 62.5)	47 (34.5, 56)	−2.5	0.01^a^^, *^
Males: females	26:24	100:89	0.01	0.9^b^
Time from onset to treatment	10 (5, 20)	7 (4, 11.5)	−2.73	0.01^b^^,*^
Underlying diseases (Yes: No)	18:32	51:138	1.57	0.21^b^
Initial PTA (dB HL)	54.4 (41.3, 71.3)	68.8 (41.3, 88.8)	−2.53	0.01^a^^, *^
Final PTA (dB HL)	45.6 (35, 61.3)	32.5 (18.8, 66.9)	−3.3	0.00 ^a^^, *^
Hearing gain (dB HL)	3.8 (0, 11.3)	21.3 (8.8, 43.8)	−8.0	0.00^a^^, *^
Audiogram curve type			9.6	0.02^b^^, *^
Ascending	14 (14.0%)	28 (14.8%)		
Sloping	31 (31.0%)	41 (21.8%)		
Flat	36 (36.0%)	53 (28.0%)		
Total deafness	19 (19.0%)	67 (35.4%)		
Vertigo	21 (42.0%)	75 (39.7%)	0.08	0.76^b^
Tinnitus	43 (86%)	181 (95.8%)	6.4	0.01^b^^, *^
Aural fulness	35 (70.0%)	153 (81.0%)	2.8	0.09^b^
Blood index				
NLR	1.7 (1.2, 2.2)	1.7 (1.3, 2.1)	−0.08	0.94^a^
MLR	0.17 (0.14, 0.24)	0.17 (0.14, 0.23)	−0.4	0.69^a^
PLR	107.3 (92.5, 141.2)	112.9 (92.5, 140.6)	−0.6	0.54^a^
CRP (mg/L)	0.3 (0.06, 1.5)	0.6 (0.1, 2.0)	−0.8	0.39^a^
Fibrinogen (g/L)	2.4 (2.1, 2.7)	2.4 (2.1, 2.8)	−0.1	0.89^a^
TBIL (μmol/L)	14.8 ± 6.0	14.3 ± 5.7	0.5	0.62^c^
IBIL (μmol/L)	10.7 ± 4.3	10.4 ± 4.1	0.5	0.60^c^
SOD (U/mL)	157.8 ± 32.1	165.3 ± 32.4	−1.5	0.15^c^
Glu (mmol/L)	4.9 (4.5, 5.6)	4.9 (4.5, 5.5)	−0.2	0.84^a^
TG (mmol/L)	1.2 (0.8, 1.8)	1.4 (1.0, 2.0)	−1.93	0.05^a^
TC (mmol/L)	4.5 (3.7, 5.3)	4.6 (4.1, 5.3)	−1.1	0.29^a^
HDL (mmol/L)	1.3 ± 0.3	1.4 ± 0.3	−2.6	0.01^c^^,*^
LDL (mmol/L)	2.7 (2.1, 3.3)	2.6 (2.1, 3.1)	−0.4	0.71^a^
Hcy (μmol/L)	9.8 (7.6, 13.5)	4.0 (2.7, 5.8)	−7.2	0.00^a^^,*^
Vestibular test (+)	44 (91.7%)	175 (92.6%)	0.05	0.83^b^
The effective rate (%)	22 (44.0%)	121 (64.0%)	6.6	0.01^b^^, *^

Si-BSSNHL, simultaneous sudden sensorineural hearing loss; USSNHL, unilateral sudden sensorineural hearing loss; NLR, neutrophil lymphocyte ratio; MLR, monocyte lymphocyte ratio; PLR, platelet lymphocyte ratio; CRP, C-reactive protein; TBIL, total bilirubin levels; IBIL, indirect bilirubin; SOD, superoxide dismutase; Glu, glucose; TG, triglyceride; TC, total cholesterol; HDL, high-density lipoprotein; LDL, low-density lipoprotein; and Hcy, homocysteine. ^*^p < 0.05.

a Mann-Whitney *U* test.

b Chi-square test.

c Independent sample *t*-test.

### 3.2. Clinical data after PSM

**Figure 1:**
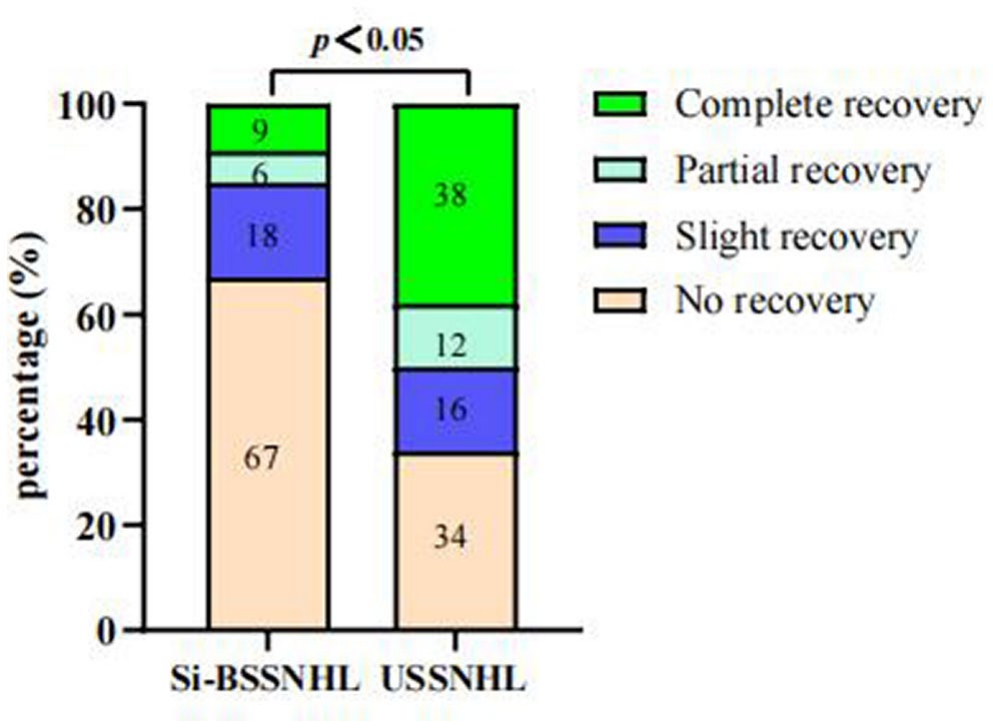
Comparisons of the efficacy between Si-BSSNHL and USSNHL after PSM. After PSM, there was a significant difference in efficacy between the Si-BSSNHL and USSNHL groups (χ^2^ = 28.5, *p* < 0.05). Si-BSSNHL, simultaneous bilateral sudden sensorineural hearing loss; USSNHL, unilateral sudden sensorineural hearing loss; PSM, propensity score matching.

There were 50 patients with USSNHL in the control group after sex and age PSM matching with Si-BSSNHL, with a median age of 48.5 years (26 males and 24 females). There were significant differences in the hearing gain, time from onset to treatment, initial PTA, final PTA, TBIL and IBIL levels, Hcy level, and the effective rate between the two groups (*p* < 0.05), as shown in Table 2. There was a significant difference in the classification of efficacy between the two groups (*p* < 0.001), as shown in Figure 1. No significant difference was found in the audiogram curve type between the two groups (*p* = 0.23), as shown in Table 2.

**Table 2:** Demographics and clinical characteristics of patients (After PSM).

variable	Si-BSSNHL (*n* = 50)	USSNHL (*n* = 50)	Statistics	Value of *p*
Time from onset to treatment	10 (5, 20)	6 (4, 10.8)	−2.6	0.01^a^^,*^
Underlying diseases (Yes: No)	18:32	10:40	3.2	0.07^b^
Initial PTA (dB HL)	54.4 (41.3, 71.3)	69.4 (45.9, 85.3)	−2.2	0.03^a^^,*^
Final PTA (dB HL)	45.6 (35, 61.3)	24.4 (17.5, 66.3)	−3.2	0.00^a^^, *^
Hearing gain (dB HL)	3.8 (0, 11.3)	22.5 (10.9, 49.4)	−6.2	0.00^a^^, *^
Audiogram curve type			4.4	0.23^b^
Ascending	14 (14.0%)	9 (18.0%)		
Sloping	31 (31.0%)	12 (24.0%)		
Flat	36 (36.0%)	13 (26.0%)		
Total deafness	19 (19.0%)	16 (32.0%)		
Vertigo	21 (42.0%)	20 (40.0%)	0.04	0.84^b^
Tinnitus	43 (86%)	48 (96.0%)	3.0	0.08^b^
Aural fulness	35 (70.0%)	37 (74.0%)	0.2	0.66^b^
Blood index				
NLR	1.7 (1.2, 2.2)	1.7 (1.3, 2.2)	−0.6	0.58^a^
MLR	0.17 (0.14, 0.24)	0.18 (0.15, 0.24)	−1.2	0.24^a^
PLR	107.3 (92.5, 141.2)	111.9 (94.5, 141.3)	−0.02	0.98^a^
CRP (mg/L)	0.3 (0.06, 1.5)	0.8 (0.3, 2.1)	−1.3	0.19^a^
Fibrinogen (g/L)	2.4 (2.1, 2.7)	2.4 (2.1, 2.8)	−0.8	0.44^a^
TBIL (μmol/L)	14.8 ± 6.0	13.5 (10.3, 16.9)	−2.3	0.02^a^^, *^
IBIL (μmol/L)	10.7 ± 4.3	9.6 (7.5, 12.7)	−2.4	0.02^a^^, *^
SOD(U/mL)	157.8 ± 32.1	147.0 (135.0, 168.3)	−1.9	0.06^a^
Glu (mmol/L)	4.9 (4.5, 5.6)	4.9 (4.5, 5.5)	−0.4	0.66^a^
TG (mmol/L)	1.2 (0.8, 1.8)	1.6 (1.1, 2.1)	−1.0	0.31^a^
TC (mmol/L)	4.5 (3.7, 5.3)	4.6 (4.3, 5.2)	−0.4	0.73^a^
HDL (mmol/L)	1.3 ± 0.3	1.4 (1.1, 1.7)	−1.5	0.13^a^
LDL (mmol/L)	2.7 (2.1, 3.3)	2.6 (2.3, 3.0)	−0.6	0.13^a^
Hcy (μmol/L)	9.8 (7.6, 13.5)	4.2 (2.5, 5.1)	−5.9	0.00^a^^, *^
Vestibular test (+)	44 (91.7%)	46 (92.0%)	0.00	0.95^b^
The effective rate (%)	22 (44.0%)	33 (66.0%)	4.9	0.03^b^^, *^

Si-BSSNHL, simultaneous sudden sensorineural hearing loss; USSNHL, unilateral sudden sensorineural hearing loss; NLR, neutrophil lymphocyte ratio; MLR, monocyte lymphocyte ratio; PLR, platelet lymphocyte ratio; CRP, C-reactive protein; TBIL, total bilirubin levels; IBIL, indirect bilirubin; SOD, superoxide dismutase; Glu, glucose; TG, triglyceride; TC, total cholesterol; HDL, high-density lipoprotein; LDL, low-density lipoprotein; and Hcy, homocysteine. ^*^p < 0.05.

a Mann-Whitney *U* test.

b Chi-square test.

### 3.3. Univariate prognostic analysis of Si-BSSNHL

The initial PTA, final PTA in the left ear of Si-BSSNHL was significantly higher than that in the right ear (Z = −3.65 and −2.43, respectively; both *p* < 0.05). Hearing gain in the left ear of Si-BSSNHL was significantly lower than that in the right ear (Z = −4.14, *p* < 0.001). No significant difference was found in the audiogram curve type between both ears of Si-BSSNHL (χ^2^ = 2.92, *p* = 0.40), as shown in Table 3. The effective rate after Si-BSSNHL treatment was 44%. Patients with Si-BSSNHL were divided into effective and ineffective groups according to their efficacy. Univariate binomial logistic regression was conducted to analyze the clinical characteristics, concomitant symptoms, combined underlying diseases (hypertension, diabetes mellitus, or coronary heart disease), audiological characteristics, vestibular function tests, and various blood index tests between the two groups. The audiogram curve type was significantly different between the different efficacy groups of patients with Si-BSSNHL (*p* = 0.01), as shown in Table 4.

**Table 3:** The PTA and audiogram curve type of both ear in the Si-BSSNHL.

Variable	Left ear (*n* = 50)	Right ear (*n* = 50)	Statistics	Value of *p*
Initial PTA (dB HL)	66.3 (50.0, 83.8)	60 (48.8, 91.3)	−3.65	0.00^a^^,*^
Final PTA (dB HL)	60 (41.3, 81.3)	56.3 (41.3, 86.3)	−2.43	0.02^a^^,*^
Hearing gain (dB HL)	3.8 (0.0, 11.3)	5(−1.3, 8.8)	−4.14	0.00^a^^,*^
Audiogram curve type			2.92	0.40^b^
Ascending	6 (12.0%)	8 (16.0%)		
Sloping	13 (26.0%)	18 (36.0%)		
Flat	22 (44.0%)	14 (28.0%)		
Total deafness	9 (18.0%)	10 (20.0%)		

Si-BSSNHL, simultaneous sudden sensorineural hearing loss. ^*^p < 0.05.

a Mann-Whitney *U* test.

b Chi-square test.

**Table 4:** Univariate logistic analysis of possible prognostic factors in Si-BSSNHL.

Variable	Effective group (*n* = 22)	Ineffective group (*n* = 28)	Statistics	Value of *p*
Age of onset	53.5 (36.7, 58.3)	56 (41, 65.8)	−1.3	0.19^a^
Time from onset to treatment	7 (5, 14.3)	16 (5.3, 28.3)	−1.4	0.16^a^
Males: Females	13 (59.1%)	13 (46.4%)	0.8	0.37^b^
Underlying diseases (Yes: No)	5: 17	7:21	0.04	0.85^b^
Vertigo	6 (27.3%)	15 (53.6%)	3.5	0.06^b^
Tinnitus	19 (86.4%)	24 (85.7%)	0.004	1.0^c^
Ear fullness	16 (72.7%)	19 (67.9%)	0.14	0.71^b^
Vestibular test (+)	19 (95%)	25 (89.2%)	0.49	0.63^c^
Audiogram curve type			11.2	0.01^b^^, *^
Ascending	10 (22.7%)	4 (7.1%)		
Sloping	7 (15.9%)	24 (42.9%)		
Flat	19 (43.2%)	17 (30.4%)		
Total deafness	8 (18.2%)	11 (19.6%)		
Deafness degree			0.8	0.86^b^
Mild	6 (13.6%)	8 (14.3%)		
Moderate	19 (43.2%)	24 (42.9%)		
Severe	12 (27.3%)	12 (21.4%)		
Profound	7 (15.9%)	12 (21.4%)		
Blood index				
NLR	1.6 (1.2,2.1)	1.9 (1.2,2.3)	−0.6	0.58^a^
MLR	0.17 (0.14,0.23)	0.18 (0.15,0.25)	−0.6	0.55^a^
PLR	103 (93.2, 130.0)	111 (90.9, 148.5)	−0.5	0.59^a^
CRP (mg/L)	0.2 (0.05, 0.7)	0.4 (0.06, 2.1)	−0.8	0.43^a^
Fibrinogen (g/L)	2.4 (2.2, 2.7)	2.3 (2.0, 2.9)	−0.7	0.48^a^
TBIL (μmol/L)	16.6 ± 7.0	13.4 ± 4.8	1.9	0.06^d^
IBIL (μmol/L)	11.9 ± 4.9	9.8 ± 3.6	1.8	0.07^d^
SOD (U/mL)	165.5 (141.8, 183.3)	155.5 (122.5, 172.8)	−1.7	0.09^a^
Glu (mmol/L)	5.0 (4.4, 5.8)	5.0 (4.5, 5.3)	−0.2	0.88^a^
TG (mmol/L)	1.0 (0.7, 1.5)	1.4 (0.9, 1.9)	−1.8	0.06^a^
TC (mmol/L)	4.4 (3.4, 5.2)	4.6 (4.0, 5.4)	−0.9	0.33^a^
HDL (mmol/L)	1.3 ± 0.3	1.3 ± 0.3	0.4	0.66^d^
LDL (mmol/L)	2.2 (1.9, 3.4)	2.9 (2.2, 3.3)	−1.3	0.18^a^
Hcy (μmol/L)	9.9 (4.0, 13.3)	10.1 (8.4, 14.7)	−0.7	0.52^a^

Si-BSSNHL, simultaneous sudden sensorineural hearing loss; CRP, C-reactive protein; TBIL, total bilirubin levels; IBIL, indirect bilirubin; SOD, superoxide dismutase; Glu, glucose; TG, triglyceride; TC, total cholesterol; HDL, high-density lipoprotein; LDL, low-density lipoprotein; and Hcy, homocysteine. ^*^p < 0.05.

a Mann-Whitney *U* test.

b Chi-square test.

c Fisher’s test.

d Independent sample *t*-test.

### 3.4. Multivariate prognostic analysis

**Figure 2:**
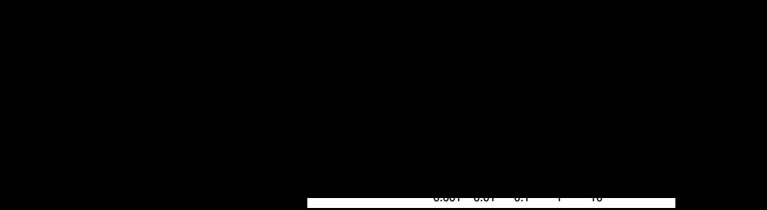
Multivariate logistic regression analysis for Si-BSSNHL. **(A)** Left ear; **(B)** Right ear; TBIL, Total bilirubin level; TG, Triglyceride; SOD, superoxide dismutase; and Si-BSSNHL, simultaneous bilateral sudden sensorineural hearing loss. ^*^p < 0.05.

Parameters that yielded a value of *p* < 0.1 in the univariate logistic regression analysis were included in the multivariate analysis for prognosis of Si-BSSNHL, such as vertigo, TBIL, IBIL, TG, SOD, and audiogram curve type. Collinearity diagnosis was made for independent variables at *p* < 0.1 prior to inclusion, and collinearity was found for TBIL and IBIL; therefore, the final variables included in the multivariable logistic regression analysis included vertigo, TBIL, TG, SOD, and audiogram curve type. Due to the differences in the audiogram curve type of the right and left ears of patients with Si-BSSNHL, multivariate analysis was performed separately for the left and right ears, as shown in Figure 2. Multivariate logistic regression analysis revealed that the sloping type (95% confidence interval, 0.006–0.549, *p* = 0.013) was linked to the efficacy of the right ear in Si-BSSNHL.

## 4. Discussion

Simultaneous bilateral sudden sensorineural hearing loss has an acute onset and unclear etiological mechanisms; it is currently a diagnosis of exclusion having poor treatment efficiency, seriously impairing quality of life, and endangering patients’ lives. Due to limitations in prevalence and data completeness, previous studies on BSSNHL have only reported a small number of cases, and most studies have analyzed clinical features and treatment efficacy; however, only a few distinctions have been made between Si-BSSNHL and Se-BSSNHL, or have been made in the post-PSM analysis for age and sex (5–8). Only Bing et al. analyzed Si-BSSNHL and Se-BSSNHL for classification and matching but did not conduct an analysis of prognostic risk factors (4). Although previous studies have analyzed the correlation between SSNHL and various blood parameters, such as prothrombotic states, metabolic parameters, inflammatory states, immunological factors, and oxidative stress, few analyses have been performed on Si-BSSNHL (9–11). The etiopathogenesis of Si-BSSNHL remains unclear due to the lack of research on the condition.

Hypertension, diabetes mellitus, and hyperlipidemia are risk factors for SSNHL’s poor prognosis (10, 12), and abnormal thyroid function has been correlated with the development of SSNHL (13). Meanwhile, the onset of hypertension and diabetes is closely associated with age, and blood indices such as bilirubin and high-density lipoprotein are also correlated with sex and age (14, 15). Abnormalities in thyroid function are most common in women (16). Therefore, the effects of age, sex, and associated confounding factors in this study can be excluded using PSM for the case and control groups. The prevalence of vertigo and aural fullness between the two groups prior to and following PSM matching was not significantly different. The prevalence of tinnitus in the case group (86%) was significantly lower than that in the control group (95.8%) before PSM, while this disparity vanished after PSM. Vestibular function abnormality was a poor prognostic factor for Si-BSSNHL. Previous studies have reported a higher rate of vestibular function abnormality in patients with BSSNHL than those with USSNHL (7), but no significant difference was found in the prevalence of vertigo between the Si-BSSNHL and USSNHL groups (4). In the present study, there was no significant difference in the prevalence of vertigo or vestibular function abnormality between the Si-BSSNHL and USSNHL groups before and after PSM. This may be since the BSSNHL in previous studies included both Si-BSSNHL and Se-BSSNHL, and different study participants may have caused differences in the results. In addition, the lower rate of vestibular function abnormality was also supported by the significantly lower initial PTA in the Si-BSSNHL group before and after PSM along with mild deafness compared to the USSNHL group. However, the present results demonstrated that patients with Si-BSSNHL had a lower hearing gain, treatment effective rate, and poorer prognosis than those with USSNHL, which is consistent with previous results (5, 6). Although there was no statistical difference in the distribution of audiogram curve type between the two groups after PSM, Si-BSSNHL was more common, with flat (36%) and sloping types (31%). While the prognosis of mid-high-frequency hearing loss is worse than that of other audiogram curve types, the prognosis of Si-BSSNHL is worse than that of USSNHL. This also suggests that Si-BSSNHL and USSNHL may have different pathogeneses; hence, despite receiving the same treatment regimens, their prognoses differ. In addition, the fact that Si-BSSNHL patients had significantly longer time from onset to treatment than USSNHL patients in this study may also influence prognosis.

The pathological mechanism underlying Si-BSSNHL is unclear, but a systemic chronic inflammatory state, prethrombotic state, and metabolic factors may be involved. CRP, NLR, MLR, and PLR can be used as reliable and convenient indicators to detect systemic chronic inflammation and coagulation status, which are associated with the prognosis of SSNHL (11, 17). Herein, we observed no significant differences in NLR, MLR, PLR, and CRP between Si-BSSNHL and USSNHL. Further univariate and multivariable analysis of Si-BSSNHL did not find any correlation between these indicators and the prognosis of Si-BSSNHL. This suggests that the systemic chronic inflammatory response and coagulation status may not be specific to the pathogenesis of Si-BSSNHL. Dyslipidemia, such as abnormalities in TG, TC, HDL, LDL, and other indicators, can be associated with the degree of hearing loss and prognosis of SSNHL by causing blood stagnation, blood flow deceleration, and lipid deposition, resulting in impaired microcirculation in the inner ear (18). In the present study, there were no significant differences between the Si-BSSNHL and USSNHL groups in any lipid metabolic indices, although the HDL levels in the Si-BSSNHL group were significantly lower than those in the USSNHL group before matching. HDL differs by sex, age, and race (14), therefore, the differences between the groups disappeared after PSM was performed. Hcy is a risk factor for vascular injury. An abnormal increase in Hcy level causes vascular endothelial dysfunction, decreased vascular flexibility, microcirculatory dysfunction, and ultimately leads to ischemic and hypoxic damage to the cochlea (19). Additionally, high Hcy level may be a high-risk factor for the development of SSNHL (9). Our results showed that Si-BSSNHL patients had significantly higher Hcy levels before and after PSM than the USSNHL group. It has been hypothesized that Hcy plays a role in the onset of inner ear microcirculatory dysfunction in Si-BSSNHL. Bilirubin is an important vascular protective factor with anti-inflammatory, antioxidant, and vasodilatory effects (20), and its level is correlated with age, sex, and oxidative stress (15). This study also showed that TBIL and IBIL levels were significantly higher in Si-BSSNHL patients than in matched USSNHL patients, although both were within the normal ranges. This suggests that Si-BSSNHL causes more severe oxidative stress damage than USSNHL does. This provides a theoretical basis for the rational clinical application of antioxidative stress drugs.

Simultaneous bilateral sudden sensorineural hearing loss has a worse prognosis than USSNHL, as reported in previous studies (5, 8). This may be due to the fact that Si-BSSNHL has distinct pathophysiologic mechanisms, possibly accounted for by an underlying systemic disease (8). Given that Si-BSSNHL has a worse prognosis, this study further analyzed the relevant factors affecting the prognosis of Si-BSSNHL. In the univariate logistic analysis, the audiogram curve type between the effective and ineffective Si-BSSNHL groups was significantly different. Multivariable logistic regression analysis of Si-BSSNHL revealed that only the sloping type had a significant effect on right ear efficacy, which was an independent risk factor for poor prognosis in the right ear of Si-BSSNHL, and the prevalence of vertigo, TBIL, TG, and SOD levels were not associated with prognosis. The pathogenesis of various audiogram types varies, and sloping-type hearing loss is often associated with cochlear base transmural cell damage, which usually has a poor prognosis (1). However, further sample size expansion and analysis of related mechanisms are required to account for the disparity in effectiveness between the right and left ears. The time from onset to treatment, the degree of deafness, and audiogram curve type are usually considered to be associated with the treatment outcome of patients with USSNHL. In this study, however, only the audiogram curve type was associated with the prognosis of Si-BSSNHL. This may be due to the various number of cases, inclusion and exclusion criteria, or efficacy evaluation criteria of different studies.

In summary, to better analyze the differences in clinical characteristics between Si-BSSNHL and USSNHL, this study applied PSM to exclude the effects of sex, age and possible confounding factors associated with sex and age. After PSM, Si-BSSNHL had mild deafness, but the degree of oxidative stress damage and inner ear microcirculation involvement was more severe than that in USSNHL, with worse prognosis. The audiogram curve type was closely related to the prognosis of Si-BSSNHL, with the sloping type being an independent risk factor for prognosis in the right ear.

## Data availability statement

The raw data supporting the conclusions of this article will be made available by the authors, without undue reservation.

## Ethics statement

The studies involving human participants were reviewed and approved by Medical Ethics Committee of Shandong Provincial ENT Hospital. Written informed consent for participation was not required for this study in accordance with the national legislation and the institutional requirements.

## Author contributions

YW and MW designed the study and wrote the manuscript. WX, XS, KL, and FD performed the research and analyzed the data. HW funded the research. All authors contributed to the article and approved the submitted version.

## Funding

This work was supported by the Major Fundamental Research Program of the Natural Science Foundation of Shandong Province, China (ZR2021ZD40), the Taishan Scholars Program of Shandong Province (ts20130913), and the Key Technology Research and Development Program of Shandong (2019GSF108248).

## Conflict of interest

The authors declare that the research was conducted in the absence of any commercial or financial relationships that could be construed as a potential conflict of interest.

## Publisher’s note

All claims expressed in this article are solely those of the authors and do not necessarily represent those of their affiliated organizations, or those of the publisher, the editors and the reviewers. Any product that may be evaluated in this article, or claim that may be made by its manufacturer, is not guaranteed or endorsed by the publisher.

## References

[1] Herrera M García Berrocal JR García Arumí A Lavilla MJ Plaza G. Grupo de Trabajo de la Comisión de Audiología de la SEORL. Update on consensus on diagnosis and treatment of idiopathic sudden sensorineural hearing loss. Acta Otorrinolaringol Esp. (2019) 70:290–300. doi: 10.1016/j.otorri.2018.04.010 30093087

[2] Schreiber BE Agrup C Haskard DO Luxon LM. Sudden sensorineural hearing loss. Lancet. (2010) 375:1203–11. doi: 10.1016/S0140-6736(09)62071-7 20362815

[3] Oh JH Park K Lee SJ Shin YR Choung YH. Bilateral versus unilateral sudden sensorineural hearing loss. Otolaryngol Head Neck Surg. (2007) 136:87–91. doi: 10.1016/j.otohns.2006.05.015 17210340

[4] Bing D Wang DY Lan L Zhao LD Yin ZF Yu L. Comparison between bilateral and unilateral sudden sensorineural hearing loss. Chin Med J. (2018) 131:307–15. doi: 10.4103/0366-6999.223843, PMID: 29363646 PMC5798052

[5] Xenellis J Nikolopoulos TP Stavroulaki P Marangoudakis P Androulakis M Tsangaroulakis M. Simultaneous and sequential bilateral sudden sensorineural hearing loss: are they different from unilateral sudden sensorineural hearing loss? ORL J Otorhinolaryngol Relat Spec. (2007) 69:306–10. doi: 10.1159/000107435, PMID: 17703107

[6] Akil F Yollu U Yilmaz M Yener HM Mamanov M Inci E. Simultaneous idiopathic bilateral sudden hearing loss—characteristics and response to treatment. Braz J Otorhinolaryngol. (2017) 84:95–101. doi: 10.1016/j.bjorl.2016.12.003, PMID: 28214147 PMC9442867

[7] Chen YH Young YH. Bilateral simultaneous sudden sensorineural hearing loss. J Neurol Sci. (2016) 362:139–43. doi: 10.1016/j.jns.2016.01.029, PMID: 26944135

[8] Elias TGA Monsanto RDC Jean LS de Souza LSR Penido NO. Bilateral sudden sensorineural hearing loss: a distinct phenotype entity. Otol Neurotol. (2022) 43:437–42. doi: 10.1097/MAO.0000000000003489, PMID: 35239621

[9] Passamonti SM Di Berardino F Bucciarelli P Berto V Artoni A Gianniello F. Risk factors for idiopathic sudden sensorineural hearing loss and their association with clinical outcome. Thromb Res. (2015) 135:508–12. doi: 10.1016/j.thromres.2015.01.001, PMID: 25619439

[10] Fasano T Pertinhez TA Tribi L Lasagni D Pilia A Vecchia L. Laboratory assessment of sudden sensorineural hearing loss: a case-control study. Laryngoscope. (2017) 127:2375–81. doi: 10.1002/lary.26514, PMID: 28224621

[11] Doo JG Kim D Kim Y Yoo MC Kim SS Ryu J. Biomarkers suggesting favorable prognostic outcomes in sudden sensorineural hearing loss. Int J Mol Sci. (2020) 21:7248. doi: 10.3390/ijms21197248, PMID: 33008090 PMC7583026

[12] Lin CF Lee KJ Yu SS Lin YS. Effect of comorbid diabetes and hypercholesterolemia on the prognosis of idiopathic sudden sensorineural hearing loss. Laryngoscope. (2016) 126:142–9. doi: 10.1002/lary.25333, PMID: 25945947

[13] Sun XM Zhuang SM Xiao ZW Luo JQ Long Z Lan LC. Autoimmune thyroiditis in patients with sudden sensorineural hearing loss. Laryngoscope Investig Otolaryngol. (2022) 7:571–7. doi: 10.1002/lio2.755, PMID: 35434320 PMC9008166

[14] von Mühlen D Langer RD Barrett-Connor E. Sex and time differences in the associations of non-high-density lipoprotein cholesterol versus other lipid and lipoprotein factors in the prediction of cardiovascular death (the rancho Bernardo study). Am J Cardiol. (2003) 91:1311–5. doi: 10.1016/s0002-9149(03)00319-9, PMID: 12767422

[15] Gazzin S Vitek L Watchko J Shapiro SM Tiribelli C. A novel perspective on the biology of bilirubin in health and disease. Trends Mol Med. (2016) 22:758–68. doi: 10.1016/j.molmed.2016.07.004, PMID: 27515064

[16] Tsai YT Chang IJ Hsu CM Yang YH Liu CY Tsai MS. Association between sudden sensorineural hearing loss and preexisting thyroid diseases: a nationwide case-control study in Taiwan. Int J Environ Res Public Health. (2020) 17:834. doi: 10.3390/ijerph17030834, PMID: 32013113 PMC7037331

[17] Seo YJ Jeong JH Choi JY Moon IS. Neutrophil-to-lymphocyte ratio and platelet-to-lymphocyte ratio: novel markers for diagnosis and prognosis in patients with idiopathic sudden sensorineural hearing loss. Dis Markers. (2014) 2014:702807. doi: 10.1155/2014/702807, PMID: 24891755 PMC4033535

[18] Shao M Xiong G Xiang G Xu S Zhang L. Correlation between serum lipid and prognosis of idiopathic sudden sensorineural hearing loss: a prospective cohort study. Ann Transl Med. (2021) 9:676. doi: 10.21037/atm-21-907, PMID: 33987374 PMC8106097

[19] Lai WK Kan MY. Homocysteine-induced endothelial dysfunction. Ann Nutr Metab. (2015) 67:1–12. doi: 10.1159/000437098 26201664

[20] Bing D Wang DY Lan L Zhao LD Yin ZF Yu L. Serum bilirubin level as a potential marker for the hearing outcome in severe-profound bilateral sudden deafness. Otol Neurotol. (2019) 40:728–35. doi: 10.1097/MAO.0000000000002287, PMID: 31135669 PMC6594721

